# Immunogenicity and immunomodulatory effects of the human chondrocytes, hChonJ

**DOI:** 10.1186/s12891-017-1547-8

**Published:** 2017-05-18

**Authors:** Chae-Lyul Lim, Yeon-Ju Lee, Jong-Ho Cho, Heonsik Choi, Bumsup Lee, Myung Chul Lee, Sujeong Kim

**Affiliations:** 1 Institute of BioInnovation Research, Kolon Life Science, Inc., Gasan-dong, Geumcheon-gu, Seoul Korea; 2 Department of Orthopaedic Surgery, Seoul National University College of Medicine, Seoul, Korea; 3 Present Address: T Cell Therapy Unit, Eutilex Research Institute of Biomedicine, Gasan-dong, Geumcheon-gu, Seoul Korea

**Keywords:** Allogeneic, Chondrocyte, Immunogenicity, Immunomodulation, PD-L1, PD-L2

## Abstract

**Background:**

Invossa™ (TissueGene-C) is a cell and gene therapy for osteoarthritis. It is composed of primary human chondrocytes (hChonJ cells) and irradiated human chondrocytes modified to express TGF-β1 (hChonJb#7 cells). The hChonJ cells were isolated from a polydactyly donor, and TGF-β1 cDNA was delivered to the cells, generating hChonJb#7 cells. Since the cells are allogeneic, the concern of immune response against cells has been raised. In this study, we investigated the immunogenicity of allogenic human chondrocyte, hChonJ cells.

**Methods:**

The immunological properties of hChonJ cells were investigated through the analysis of surface marker expression and the effect on allogeneic T cell proliferation. Flow cytometry and RT-PCR analysis were performed to analyze the surface marker expression related to immune response, such as major histocompatibility complex (MHC) class I, class II, T cell co-stimulatory molecules and T cell co-inhibitory molecules. A mixed lymphocyte reaction (MLR) was conducted to evaluate how allogeneic T cells would respond to hChonJ cells.

**Results:**

We observed that hChonJ cells did not express MHC class II and T cell co-stimulatory molecules, but expressed T cell co-inhibitory molecule PD-L2. IFN-γ treatment induced the expression of PD-L1, and up-regulated the expression of PD-L2. Also, we observed that hChonJ cells did not stimulate T cell proliferation from a MHC-mismatched donor. Further, they could suppress the proliferation of activated T cells. We also observed that the blockade of PD-L1 and/or PD-L2 with specific neutralizing antibody could lead to the restoration of allo-reactive T cell proliferation.

**Conclusions:**

We showed that hChonJ cells were not immunogenic but immunosuppressive, and that this phenomenon was mediated by co-inhibitory molecules PD-L1 and PD-L2 on hChonJ cells in a contact-dependent manner.

**Electronic supplementary material:**

The online version of this article (doi:10.1186/s12891-017-1547-8) contains supplementary material, which is available to authorized users.

## Background

Invossa™ (﻿TissueGene-C) is a cell and gene medicine for osteoarthritis [1–3]. It is a mixture of primary human chondrocytes (hChonJ cells) and irradiated human chondrocytes modified to express TGF-β1 (hChonJb#7 cells) by the ratio of 3:1, and is administered into a knee joint of patients. The components of Invossa™, hChonJ and hChonJb#7 cells are allogeneic. The hChonJ cells were isolated from a cartilage of a 1-year-old female polydactyly donor and expanded in a monolayer culture. TGF-β1 cDNA was transferred to the hChonJ cells using retroviral vector to generate hChonJb#7 cells. Therefore, there has been a concern that these cells could induce immune responses when injected to patient’s joints. To address this question, the efficacy and safety of Invossa™ was evaluated in several animal models [4–6]. Invossa™ showed efficacy in xenogeneic animals, and no adverse reaction related to Invossa™ was observed. Based on these data, clinical trials have been initiated. Up until now, Invossa™ has been administered more than 200 patients in several clinical trials, but no serious adverse events related to the cell components have been reported [7–10]. However, no scientific evidence that Invossa™ does not induce immune response has been provided so far.

Clinical experiences over the last 30 years have shown that osteochondral allograft transplantation does not elicit immune response [11, 12]. In addition, there are a volume of reports showing that transplanted allogeneic chondrocytes are not rejected. Transplanted osteochondral graft expresses donor MHC molecules, the primary target of the immune response to allogeneic tissues. Usually, transplanted tissue is rejected when the recipient T cells recognize donor tissue as non-self, and this process is mediated by MHC molecules present on the surface of donor cells. However, in osteochondral allografts, a host immune response against chondrocytes has not been reported. It is thought that the environmental characteristics of articular cartilage such as avascular and alymphatic extracellular matrix surrounding them plays a role. The extracelluar matrix can shield the MHC molecules from recognition by host cells; thereby protecting the chondrocytes from host immune responses [13, 14]. The results are same with xenogeneic transplantation. When human neocartilage was transplanted into surgical defects created in the knee joint in genetically unrelated recipients, it was not rejected [15, 16]. Human juvenile chondrocytes present in bioengineered neocartilage lack cell surface markers required for immune responses. They do not induce alloantigen specific proliferative immune responses in vitro, and they actively suppress the proliferation of activated T cells in a cell to cell contact-dependent manner. These results suggest that the immunosuppressive properties of chondrocytes may help immune evasion of allograft [17].

As described previously, the hChonJ cells were derived from very young donor (1 year-old), therefore it is possible that hChonJ cells would share the immunosuppressive properties of human juvenile chondrocytes. However, hChonJ cells were cultured in monolayer and dedifferentiated, so we cannot be sure whether they preserve the properties of juvenile chondroctyes. In this study, we investigated the cell surface marker expression related to immune reaction and immunological properties of hChonJ cells, the live cell component of Invossa™.

## Methods

### Materials

Dulbecco’s Modified Eagle’s Medium (DMEM) with high glucose, RPMI-1640, 1x antibiotic-antimycotic solution, and trypsin–EDTA solution were purchased from Gibco (CA, USA). Fetal bovine serum (FBS) was purchased from Hyclone (UT, USA). Antibodies for FACS analysis, including Isotype IgG1, IgG2a, HLA-ABC (MHC class I), HLA-DR (MHC-class II), CD80 (B7-1), CD86 (B7-2), CD137L (4-1BBL), CD152 (CTLA-4), CD252 (OX40L), CD273 (PD-L2), CD274 (PD-L1), CD278 (ICOS), CD279 (PD-1) and B7H4 were purchased from BD Biosciences (CA, USA). Also, CD274 (PD-L1) and CD273 (PD-L2) antibodies were purchased from eBioscience (CA, USA). The detailed information of used antibodies was summarized in Table 1. Recombinant interferon (IFN)-γ was purchased from Peprotech (NJ, USA), isopropyl alcohol, mitomycin C and phytohemagglutinin were purchased from Sigma Chemical (MO, USA) and ^3^H-thymidine was purchased from American Radiolabeled Chemicals (MO, USA). Other materials included SuperScript™III First-Strand Synthesis System and Trizol from Invitrogen (CA, USA), PCR reagents from TAKARA Bio (Shiga, Japan) and Ficoll-Paque PLUS from GE Healthcare (Sweden).

**Table 1:** Antibody clones and isotypes

Antibody name	Clone	Isotype
HLA-ABC	G46-2.6	Mouse IgG_1_, kappa
HLA-DR	G46-6	Mouse IgG_2a_, kappa
CD44	515	Mouse IgG_1_, kappa
CD73	AD2	Mouse IgG_1_, kappa
CD80	L307.4	Mouse IgG_1_, kappa
CD86	FUN-1	Mouse IgG_1_, kappa
CD90	5E10	Mouse IgG_1_, kappa
CD105	SN6	Mouse IgG_1_, kappa
CD137L (4-1BBL)	C65-485	Mouse IgG_1_, kappa
CD151	14A2.H1	Mouse IgG_1_, kappa
CD152 (CTLA-4)	BNI3	Mouse IgG_2a_, kappa
CD252 (OX40L)	ik-1	Mouse IgG_1_, kappa
CD273 (PD-L2)	MIH18	Mouse IgG_1_, kappa
CD274 (PD-L1)	MIH1	Mouse IgG_1_, kappa
CD278 (ICOS)	DX29	Mouse IgG_1_, kappa
CD279 (PD-1)	MIH4	Mouse IgG_1_, kappa
Mouse IgG_1_, kappa isotype control	X40	Mouse IgG_1_, kappa
Mouse IgG_2a_, kappa isotype control	X39	Mouse IgG_2a_, kappa

### Cell culture

hChonJ cells were cultured at Wuxi AppTec (Philadelphia, PA, USA) in accordance with Good Manufacturing Practices in DMEM supplemented with 10% heat-inactivated FBS and antibiotic-antimycotic solution. As the positive control for MHC class I, II, CD80 and CD86 molecule, Raji cells were cultured in RPMI-1640 supplemented with 10% heat-inactivated FBS and antibiotic-antimycotic solution. For IFN-γ treatment, hChonJ cells were incubated in the presence of 300 IU/mL of INF-γ for 96 h.

### Flow cytometric analyses

The expression of immune-related cell surface markers was analyzed by flow cytometry using the FACSCanto™ II (BD Biosciences). Cells were washed with PBS and incubated with respective monoclonal antibody (mAb) or the isotype control for 30 min in the dark at 4 °C. Then, cells were washed and resuspended in PBS, and analyzed on the FACSCanto™ II using FACS DIVA software. The fluorescence intensity of each antibody was compared with that of the isotype control and represented as a histogram of measurements taken from each cell. These experiments were performed three times independently.

### mRNA analyses of cell surface molecules and indoleamine 2,3-dioxygenase (IDO)

The mRNA expression of cell surface molecules and IDO was assessed using a reverse-transcription polymerase chain reaction (RT-PCR). All primers were ordered from Macrogen (Seoul, Korea). The sequences of primers are shown in Table 2. Total RNA was extracted using TriZol according to the manufacturer’s instructions. cDNA was synthesized by using SuperScript™ III First-Strand Synthesis System. The procedures were as follows: 1 μg of total RNA, 1 μL of 50 μM oligo(dT) primer, and 1 μL of 10 mM dNTP mix adjusted to a total volume of 10 μL with DEPC-treated water. Mixtures were incubated for 5 min at 65 °C; then, 2 μL of 10x RT buffer, 4 μL of 25 mM MgCl_2_, 2 μL of 0.1 M DTT, 1 μL of RNaseOUT™, and 1 μL of SuperScript™ III RT were added to each tube. The reaction was run in a BioRad thermocycler for 50 min at 50 °C and 5 min at 85 °C; then, PCR was performed using 2 μL of cDNA, 2 μL of 100 μM gene specific primers, 10x PCR buffer, 2.5 mM dNTP mix, 5 U/μL Taq polymerase adjusted to a total volume of 20 μL with distilled water. PCR conditions included initial denaturation step at 95 °C for 5 min, followed by 40 cycles of denaturation at 95 °C for 30 s, annealing for 30 s and extension at 72 °C for 1 min. Amplification was finished with extension at 72 °C for 5 min. A housekeeping gene β-actin was used as an internal control.

**Table 2:** PCR primers

Gene name	Sequence	Amplicon size (bp)
HLA-ABC (MHC class I)	Sense, 5′-GATTCTCCCCAGACGCCGAG-3′ Antisense, 5′-CCTGGGCACTGTCACTGCTT-3′	1082
CD274 (PD-L1)	*Sense, 5′-CTACCCCAAGGCCGAAGTCA-3′* *Antisense, 5′-CCCAGAATTACCAAGTGAGTCC-3′*	*258*
CD273 (PD-L2)	Sense, 5′-ACCCTGGAATGCAACTTTGACACT-3′ Antisense, 5′-ACTTGGACTTGAGGTATGTGGAACGA-3′	167
CD80	Sense, 5′-CATCACGGAGGGTCTTCTAC-3′ Antisense, 5′-AGGATCTTGGGAAACTGTTGT-3′	710
CD86	Sense, 5′-TGCAAACTCTCAAAACCAAAG-3′ Antisense, 5′-AAAACACGCTGGGCTTCATCA-3′	815
IDO	Sense, 5′-AGGCAACCCCCAGCTATCAGAC-3′ Antisense, 5′-TCAGGGAGACCAGAGCTTTCACAC-3′	314
β-actin	Sense, 5′-GCTCGTCGTCGACAACGGCTC-3′ Antisense, 5′-CAAACATGATCTGGGTCATCTTCTC-3′	329

The expression of IDO mRNA was also assessed using a quantitative reverse transcripion-polymerase chain reaction (qRT-PCR). All primers were ordered from Macrogen and the sequences of primers are shown in Table 3. β-actin was used to normalize for IDO mRNA expression. cDNA was synthesized as described above for RT-PCR of IDO mRNA. Quantitative reverse transcriptase-polymerase chain reaction (qRT-PCR) was performed with 2 μL of synthesized cDNA by using the SYBR premix EX taq (TAKARA Bio) according to the manufacturer’s instruction. The reactions were run on a Rotor-gene Q system (Qiagen, Hilden, Germany) in triplicate. PCR conditions included initial denaturation step at 95 °C for 5 min, followed by 40 cycles of denaturation at 95 °C for 10 s, annealing at 58 °C for 15 s and extension at 72 °C for 20 s. The quantification data were analyzed with the Rotor Gene software to obtain relative IDO expression and PCR products were examined for the presence of by‑products using the melting curve analysis provided by the software.

**Table 3:** Quantitative real-time PCR primers

Gene name	Sequence
IDO	Sense, 5′- CTGGGCATCCAGCAGACT-3′ Antisense, 5′- TGAGCTGGTGGCATATATCTTCT-3′
β-actin	Sense, 5′- CAACCGCGAGAAGATGAC-3′ Antisense, 5′- GTCCATCACGATGCCAGT-3′

### Mixed lymphocyte reaction (MLR) assay

Immunogenicity of hChonJ cells was assessed using an MLR assay. In this MLR assay, we used hChonJ cells as the stimulator, and MHC-mismatched peripheral blood mononuclear cells (PBMCs) as the responder. Another MHC-mismatched PBMCs were used as the stimulator for a positive control of MLR assay. Human PBMCs were obtained by density gradient centrifugation using Ficoll-Paque™ PLUS according to the manufacturer’s instructions. Stimulator cells (hChonJ cells and PBMCs obtained from MHC-mismatched donor) were treated with 40 μg/mL of mitomycin C (MMC) to prevent cell proliferation. 1 × 10^5^ responder PBMCs were co-cultured with 1 × 10^5^ cells of MMC treated stimulator PBMCs or various numbers of MMC treated hChonJ cells (1 × 10^3^, 1 × 10^4^ and 5 × 10^4^ cells) in triplicate in round bottom 96-well plates (Nunc, Roskilde, Denmark). To confirm the mitogenic activity of responder PBMCs, the cells treated with 2 μg/mL of phytohemagglutinin (PHA) were also cultured. Cell culture for MLR assay was performed in RPMI-1640 supplemented with 10% FBS and antibiotic-antimycotic solution for 4 days at 37 °C. Eighteen hours before the end of culture, 0.5 μCi/well of ^3^H-thymidine was added to each well to measure the proliferation of responder PBMCs. Then, cells were harvested using an automated cell harvester (Tomtec, Hamden, CT), and thymidine incorporation was measured by Wallac MicroBeta-2 Scintillation Counter (PerkinElmer, Boston, MA).

### Immune suppression assays

The immune suppressive activity of hChonJ cells was assessed using an allogeneic MLR assay. To evaluate their ability to inhibit the proliferation of responder cells, hChonJ cells (1 × 10^3^, 1 × 10^4^ and 5 × 10^4^ cells/well) were added to an allogeneic MLR culture for 4 days. In addition, to evaluate whether the immune suppressive mechanism is caused by cell to cell interaction or soluble factors, assays were performed in two different culture conditions. First, hChonJ cells (1 × 10^3^, 1 × 10^4^ and 5 × 10^4^ cells/well) were added to an allogeneic MLR culture with 20 μL of antibodies against PD-L1 and/or PD-L2 to mask co-inhibitory molecules expressed on hChonJ cells. Second, allogeneic MLR cultures were separated from hChonJ cells using transwell plate with a 0.2 μm pore membrane (Nunc, MA, USA). Cells were cultured for 4 days, and 0.5 μCi/well of ^3^H-thymidine was added to each well 18 h before the end of culture.

### Statistical analysis

Statistical analysis was performed using Sigma Plot (version 13.0) software. All data are presented as the mean ± SE. Statistically significant differences among study groups were determined by one-way analysis of variance (ANOVA) and Tukey’s test was applied as a post hoc test if statistical significance was determined. Statistical significance was defined as *P* < 0.05.

## Results

### Expression of cell surface molecules on hChonJ cells

**Fig. 1:**
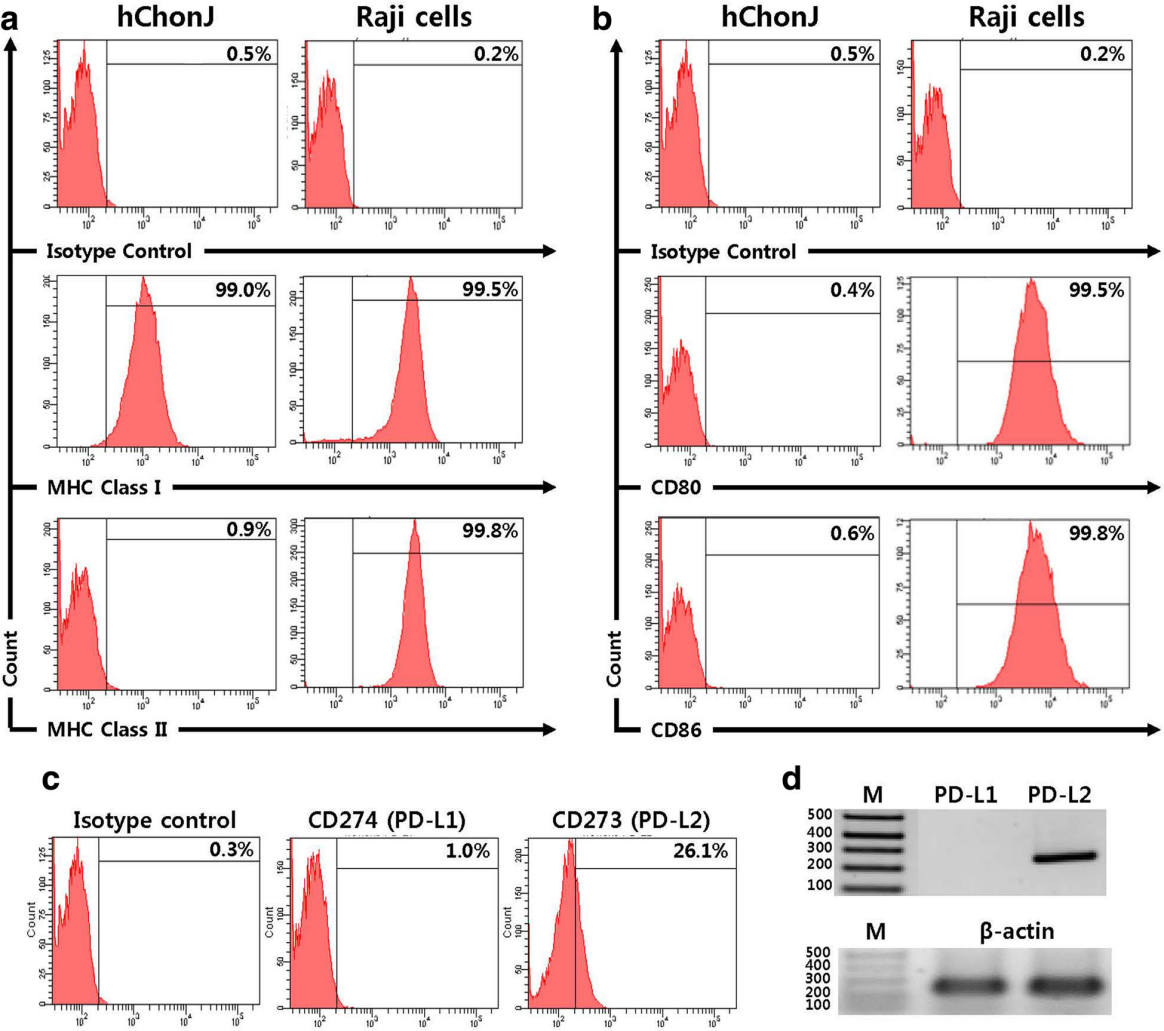
Flow cytometry analysis for immune-related surface marker on hChonJ. hChonJ cells were labeled with antibodies against immune-related surface markers, including MHC class I (HLA-ABC), MHC class II (HLA-DR), co-stimulatory molecules (CD80 and CD86), co-inhibitory molecule, (CD274 (PD-L1) and CD273 (PD-L2)) and immunoglobulin G1 isotype control. Raji cells were used as controls. hChonJ cells express MHC class I (**a**), but not MHC class II (**a**) and co-stimulatory molecules CD80, CD86 (**b**). In addition, hChonJ express low level of PD-L2, but not PD-L1 (**c**). RT-PCR confirmed the expression of PD-L2 in hChonJ (**d**). These results are the representative of at least 3 independent experiments

As the first step to evaluate the immunological property of hChonJ cells, we investigated cell surface marker expression related to immune response, such as major histocompatibility complex (MHC) class I, class II, co-stimulatory molecules and co-inhibitory molecules. Flow cytometry analysis showed that hChonJ cells expressed MHC class I (HLA-ABC), but not MHC class II (HLA-DR), which was expected because non-immune cells usually express only MHC class I (Fig. 1a). hChonJ cells did not express any of co-stimulatory molecules, such as B7-1 (CD80) and B7-2 (CD86) (Fig. 1b), whose signals are critical for T cell activation. We also measured the expression of other co-stimulatory molecules, CD137L (4-1BBL), CD252 (OX40L), CD278 (ICOS), but none of these molecules were expressed on hChonJ cells (Additional file 1: Figure S1). When the expression of co-inhibitory molecules was analyzed, CD28/CTLA-4 family molecules, B7-H4, CD152 (CTLA-4), PD-1, and PD-L1 was not detected on hChonJ cells (Additional file 1: Figure S1). However, hChonJ cells expressed low levels of PD-L2 by flow cytometry and RT-PCR (Fig. 1c, d). To observe whether the passage of hChonJ cells could impact on the cell surface marker expression, we passaged hChonJ cells in monolayer for 3 additional times, and repeated flow cytometric analysis. As a result, the expression patterns were not changed when hChonJ cells were further passaged (Additional file 2: Figure S2).

**Fig. 2:**
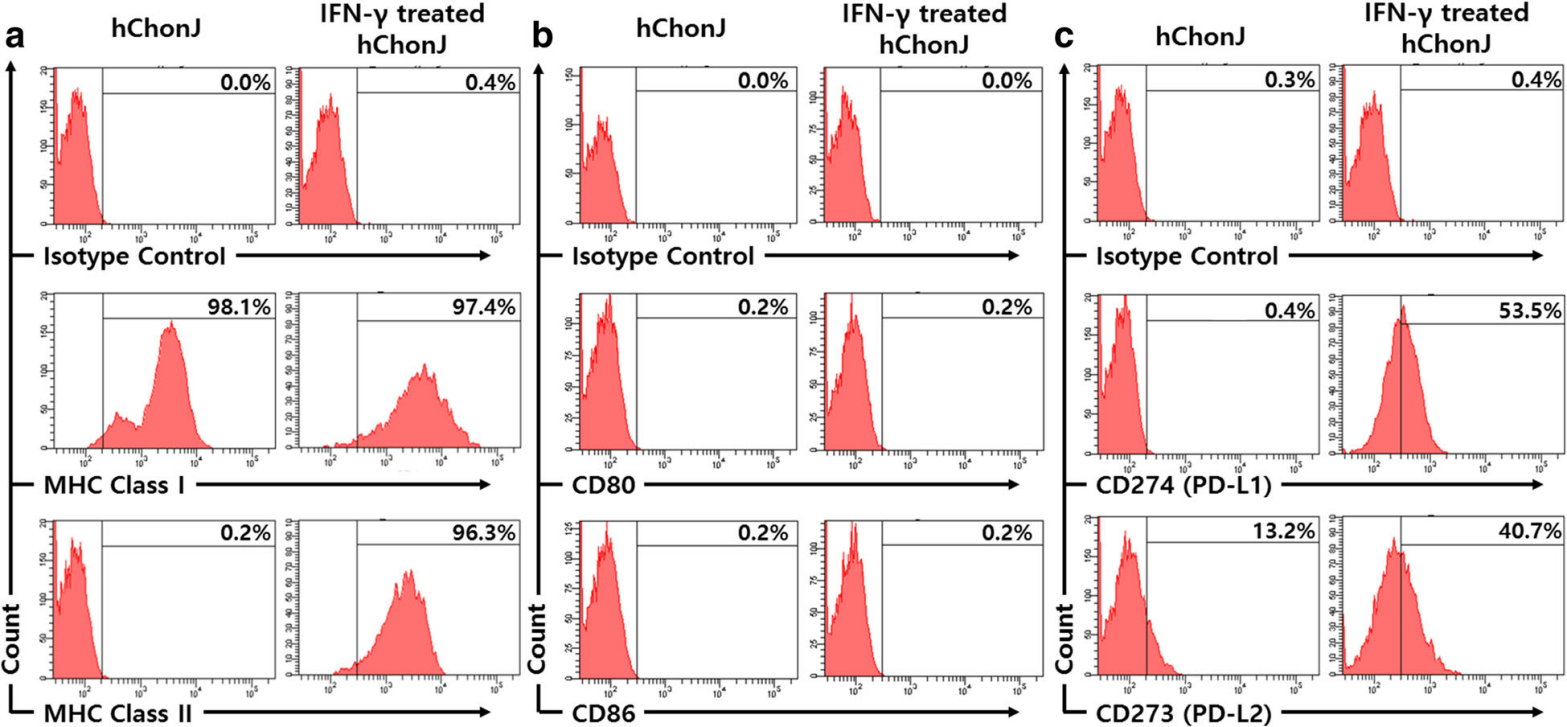
Flow cytometry analysis for the change of immune-related surface markers on IFN-γ treated hChonJ. After hChonJ cells were exposed to 300 IU/mL of IFN-γ, the cells were labeled with antibodies against immune-related surface markers, including MHC class I (HLA-ABC), MHC class II (HLA-DR), co-stimulatory molecules (CD80 and CD86), co-inhibitory molecules (PD-L1 and PD-L2) and immunoglobulin G1 isotype control. The expression of MHC class I and II molecules (**a**) was increased, but there was no change in the expression level of the co-stimulatory molecules CD80 and CD86 (**b**). In the case of co-inhibitory molecules, PD-L1 expression was increased and the expression of PD-L2 was detected which was not expressed on IFN-γ untreated hChonJ cells (**c**). These results are the representative of at least 3 independent experiments

We evaluated the changes in cell surface marker expression after culturing hChonJ cells in the presence of IFN-γ for 96 h. The expression level of MHC class I was increased by IFN-γ (Fig. 2a). MHC class II molecules were not expressed prior to IFN-γ treatment, but more than 90% of cells became MHC class II positive after IFN-γ treatment (Fig. 2a). However, there was no change in the expression of the co-stimulatory molecules, CD80 and CD86 (Fig. 2b). We observed that the expression of co-inhibitory molecules PD-L1 and PD-L2 was highly up-regulated when hChonJ cells were treated with IFN-γ (Fig. 2c).

### The immunosuppressive properties of hChonJ cells on a mixed lymphocyte reaction

**Fig. 3:**
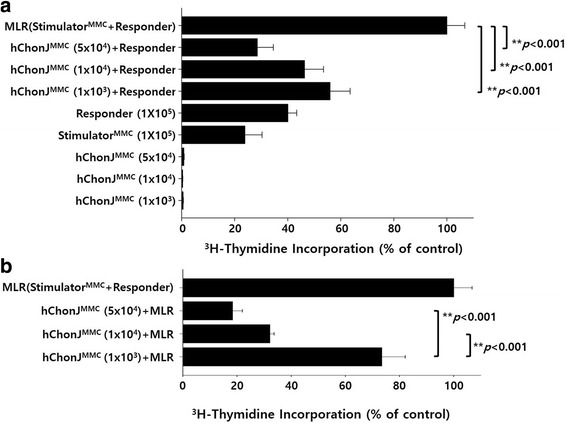
Immunogenecity and immunosuppressive effect of hChonJ. The immunogenicity and immunosuppressive effects of allogeneic hChonJ cells were assessed using an MLR assay with allogeneic PBMCs that were isolated from a MHC mismatched donor. The proliferation of responder PBMCs was measured with 0.5 μCi ^3^H-thymidine. hChonJ cells did not stimulate allogeneic T cell proliferation (**a**) and significantly suppressed allogeneic T cell proliferation in a dose-dependent fashion (**b**). Bars represented the change of ^3^H-thymidine uptake in comparison with an allogeneic MLR control (100%). Mean values from 4 independent experiments are illustrated (Mean ± SE)

In this study, we examined the immunogenicity of hChonJ cells ex vivo using MLR assays. For the reaction, we used non-dividing hChonJ cells treated with MMC as stimulator cells and allogeneic human PBMCs as responder cells, and we used non-dividing allogeneic PBMCs treated with MMC for positive response of MLR. Three doses of non-dividing hChonJ cells (1 × 10^3^, 1 × 10^4^ and 5 × 10^4^ cells) were mixed with 1 × 10^5^ allogeneic responder PBMCs. We observed that hChonJ cells did not elicit the proliferation of allogeneic responder PBMCs on day 4, while the non-dividing allogeneic stimulator PBMCs induced robust responder PBMC proliferation (Fig. 3a).

In order to investigate whether hChonJ cells can suppress alloreactive T cells, three doses of non-dividing hChonJ cells (1 × 10^3^, 1 × 10^4^ and 5 × 10^4^ cells) were added to an MLR culture. We confirmed that hChonJ cells could suppress allogeneic PBMC proliferation in a dose-dependent manner (Fig. 3b) (*p* < 0.001).

To summarize, although hChonJ cells express MHC class I molecules, they do not stimulate alloantigen-specific T cell proliferation. Also, hChonJ cells have the ability to suppress alloreactive T cells. In the following experiment, we investigated the mechanism of hChonJ cells-mediated T cell suppression.

### Mechanism of hChonJ cells-mediated immune suppression

**Fig. 4:**
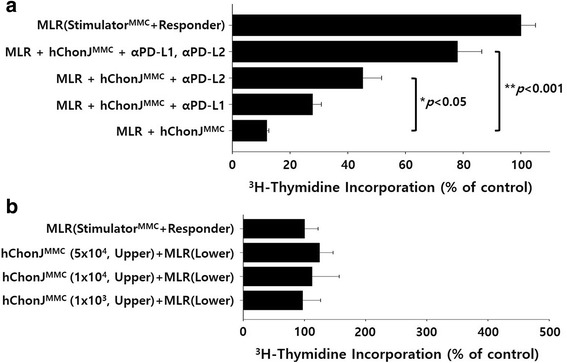
Immunosuppressive mechanism of hChonJ. In order to investigate the mechanism involved in the suppression of T cell proliferation mediated by hChonJ cells, we evaluated whether this phenomenon was dependent on a cell to cell contact or soluble molecules. To study the involvement of the cell to cell contact mechanism, antibodies against PD-L1 and/or PD-L2 were directly added to the MLR culture. The involvement of soluble molecules was studied by using a trans-well system where hChonJ (upper compartment) was physically separated from the MLR culture (lower compartment). The proliferation of responder PBMCs was measured with 0.5 μCi ^3^H-thymidine. Alloreactive T cell proliferation that was suppressed by hChonJ cells was significantly restored by treatment of PD-L1 and/or PD-L2 antibodies (**a**), but hChonJ cells did not exert their suppressive effects on MLR culture in a trans-well system (**b**). Bars represented the change of ^3^H-thymidine uptake in comparison with an allogeneic MLR control (100%). These results are the representative of 3 independent experiments (Mean ± SE)

We tested whether the inhibition of T cell proliferation by hChonJ cells was dependent on a cell to cell contact mechanism or mediated by soluble molecules. hChonJ cells expressed co-inhibitory molecules PD-L1 and PD-L2 (Fig. 2c), which are known to regulate immune response [18]. To examine if the inhibitory mechanism is caused by cell to cell contact, non-dividing hChonJ cells (5 × 10^4^ cells) were added to an MLR culture with neutralizing antibodies against PD-L1 and/or PD-L2 to mask co-inhibitory molecules expressed on hChonJ cells. Alloreactive T cell proliferation suppressed by hChonJ cells was significantly restored by the blockade of PD-L2 (*p* = 0.006), and both PD-L1 and PD-L2 (*p* < 0.001) (Fig. 4a). To examine if the inhibition is also caused by soluble molecules, we performed an MLR in a trans-well system where hChonJ cells were cultured in the upper compartment, physically separated from MLR culture in the lower compartment. We found that hChonJ cells did not exert the suppressive effects on MLR without physical contact (Fig. 4b). These results suggested that the immune suppression by hChonJ cells is mediated through a cell to cell contact not through soluble factors, and PD-L1 and PD-L2 present on hChonJ cells play an important role.

### Expression of indoleamine 2,3-dioxygenase on hChonJ cells

**Fig. 5:**
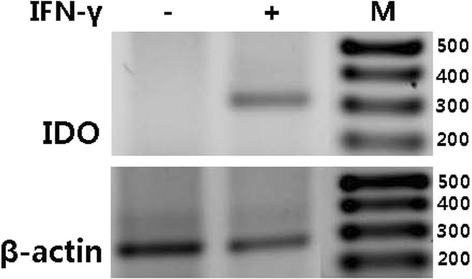
RT-PCR analysis for indoleamine 2,3-dioxygenase (IDO). IDO mRNA expression in hChonJ was analyzed by RT-PCR. The RT-PCR results showed the expression of IDO mRNA was induced by IFN-gamma treatment. Beta-actin mRNA served as an internal control

We investigated further if there are other mechanisms responsible for the immunosuppressive activity of hChonJ cells. We selected indoleamine 2,3-dioxygenase (IDO) as a candidate because it is well-known that IDO can suppress T-cell immune response by catabolizing the essential amino acid tryptophan along the kynurenine pathway from the cellular microenvironment [19–21]. In this study, we investigated whether hChonJ cells express IDO. We cultured hChonJ cells with or without IFN-gamma, and performed RT-PCR to detect IDO mRNA. As shown in Fig. 5, we observed that hChonJ cells did not express IDO mRNA under normal condition but did express IDO mRNA when they were treated with IFN-gamma. We also analyzed the expression of IDO mRNA by qRT-PCR. When hChonJ cells were treated with IFN-gamma, the Ct value of IDO mRNA was changed from baseline undetected (Ct ≥ 40) to 19.47 (SD = 0.82). This result confirmed the RT-PCR data showing that the IDO gene expression was induced by IFN-gamma treatment in hChonJ cells.

## Discussion

Invossa™ is an allogeneic cell and gene medicine for osteoarthritis, and injected to the arthritic knee joints. Articular cartilage is avascular and alymphatic, and it has a thick layer of extracellular matrix which can protect chondrocytes from immune cells. However, in osteoarthritic conditions, the immune-privileged nature of articular cartilage can be lost due to the damage to cartilage. Osteoarthritis has long been considered non-inflammatory arthropathy [22]. However, the involvement of an inflammatory component, which is marked by symptoms such as joint pain and swelling, is now well recognized. The inflammatory response occurring in the synovial membrane of osteoarthritic patients exhibits features of a T cell immune response [23]. It has been reported that CD3 positive T cells infiltrate in the synovium of osteoarthritic patients [24] and T helper cells promote disease progression of osteoarthritis [25]. Therefore, an antigen-driven immune response exists in osteoarthritic patients. T cells, with chronic inflammatory conditions, might be responsible for chondrocytes degeneration or hyaluronic acid degradation [24].

In this study, we investigated the immunogenicity of dedifferentiated allogeneic chondrocytes (hChonJ cells) and found that hChonJ cells did not induce a T cell response, but rather could suppress an immune response. First, we tested the expression of cell surface molecules on hChonJ cells and observed that they expressed MHC class I, but did not express MHC class II and co-stimulatory molecules, CD80 and CD86 (Fig. 1a, b). Interestingly, hChonJ cells expressed co-inhibitory molecule PD-L2 (Fig. 1c, d). PD-L2 expression is known to be restricted to dendritic cells and macrophages, although PD-L1 is expressed on both hematopoietic cells and non-hematopoietic cells. PD-L2 expression in non-hematopoietic cells was reported in human umbilical vein endothelial cells (HUVEC) by flow cytometry [26] and chondrocytes by RT-PCR [17]. However, this is the first report that PD-L2 was constitutively expressed on human chondrocytes at the protein level. We observed that 20 – 30% of hChonJ cells expressed PD-L2 by flow cytometry. We also analyzed the expression of cell surface molecules on TGF-β 1 overexpressing chondrocytes, and observed that there was no difference in the expression pattern between hChonJ cells and TGF-β 1 overexpressing cells (data not shown).

In addition, when hChonJ cells were exposed to IFN-gamma, the expression of PD-L1 as well as that of PD-L2 was up-regulated (Fig. 2c). Both molecules were reported to be up-regulated mainly upon exposure to IFN-γ in monocytes, dendritic cells and cancer cells, however, it was not reported in chondrocytes at the protein level [27–31]. These two co-inhibitory molecules, PD-L1 and PD-L2, can suppress activated T cells through binding with PD-1 and it is known that cancer cells use this strategy to evade host immune response. Our results indicate the possibility that hChonJ cells can suppress activated T cells via these co-inhibitory molecules.

We also tested the immunogenicity and immunosuppressive effect of hChonJ cells by using MLR assays. When hChonJ cells were co-cultured with allogeneic PBMCs, hChonJ cells did not stimulate alloantigen-specific T cell proliferation (Fig. 3a). In order to investigate whether hChonJ cells can suppress alloreactive T cells, we added hChonJ cells to allogeneic MLR culture. As shown in Fig. 3b, hChonJ cells significantly suppressed allogeneic T cell proliferation in a dose-dependent manner. We already knew that hChonJ cells express co-inhibitory molecules, PD-L1 and PD-L2, which can suppress the activated T cells. Here, we investigated whether the suppression of T cell proliferation by hChonJ cells is mediated by a cell to cell contact through PD-L1 and/or PD-L2. We used anti-PD-L1 and/or PD-L2 antibody to block its interaction with PD-1 on activated T cell. The blockade of PD-L1 and/or PD-L2 with specific antibody led to restoration of alloreactive T cell proliferation (Fig. 4a). When both anti-PD-L1 and anti-PD-L2 antibody was present, T cells proliferation was significantly restored. However, these two antibodies did not affect the allogeneic MLR by MMC-treated stimulator PBMCs (data not shown). To investigate whether soluble factors secreted by hChonJ cells are also involved in suppression of T cell proliferation, we performed an MLR in flat-bottom, trans-well system. However, in this system, hChonJ cells did not exert their suppressive effects on MLR culture (Fig. 4b). Therefore, interactions of the PD-L1, PD-L2 and PD-1, which are delivered by direct contact between hChonJ cells and allogeneic T cells, are required to weaken T cell proliferation.

We further searched other molecules involved in T cell suppression by hChonJ cells, and found out that IDO might play a role in this phenomenon. IDO is one of the major immunosuppressive molecules that inhibit T-cell immune response against alloantigens [32, 33]. It catabolizes tryptophan to kynurenine, resulting in localized depletion of tryptophan and inhibition of cell proliferation. The expression of IDO was reported in various cells. BM-MSCs can inhibit an allogeneic T cell response through the expression of IDO [34]. Also, Adkisson and the colleagues suggested IDO expression as a mechanism of neocartilage implant survival [17]. We found out that hChonJ cells also express IDO (Fig. 5). IDO expression might be involved in the immunosuppressive activity of hChonJ cells like the case of MSC or neocartilage. We did not evaluate the expression of other immunosuppressive molecules such as arginase or iNOS in ChonJ cells. This possibility should be clarified in further study.

In this report, we studied the immunogenicity of human chondrocytes obtained from one donor. The immunogenic properties observed in hChonJ cells may not be repeated in other chondrocytes obtained from other sources. This is the limit of our report, and we hope that our research will be the basis for further research.

## Conclusions

The hChonJ cells are not immunogenic and can suppress alloreactive T cell proliferation. It is known that chondrocytes have immunosuppressive effect, and has been surmised that co-inhibitory molecule are involved [16]. However, there have been no reports showing the involvement of PD-L1 and PD-L2 in the immunosuppression of chondrocytes. Here, we showed the PD-L1 and PD-L2 were involved in the immunosuppressive effect of chondrocyte through a cell to cell contact. Because of these immunological properties, clinical applications of allogeneic hChonJ cells would not induce an immune response. We have not tested the persistence of the effects on humans. However, we could deduce that hChonJ cells did not stimulate immune response in human from the fact that the efficacy of Invossa™ lasted more than a year in clinical trials.

## Additional files

Additional file 1: Figure S1. Flow cytometry analysis for immune-related surface marker on hChonJ. (JPG 216 kb)

Additional file 2: Figure S2. Flow cytometry analysis for the expression pattern of MHC class I, MHC class II, co-stimulatory molecule (CD80, CD86) and co-inhibitory molecule (PD-L1, PD-L2) on 3 additional passaged hChonJ cells. (JPG 256 kb)

## Abbreviations

IDO: Indoleamine 2,3-dioxygenase
IFN-γ: Interferon gamma
MHC: Major histocompatibility complex
MLR: Mixed lymphocyte reaction
MMC: Mitomycin C
PBMC: Peripheral blood mononuclear cell
PD-L1: Programmed death-ligand 1
PD-L2: Programmed death-ligand 2
PHA: Phytohemagglutinin
TGF-β1: Transforming growth factor beta 1
