# Supplementary figures and images for: Immunogenicity and immunomodulatory effects of the human chondrocytes, hChonJ
Source: BMC Musculoskelet Disord. 2017 May 18;18:199. doi: 10.1186/s12891-017-1547-8 (PMC5437658; doi:10.1186/s12891-017-1547-8)

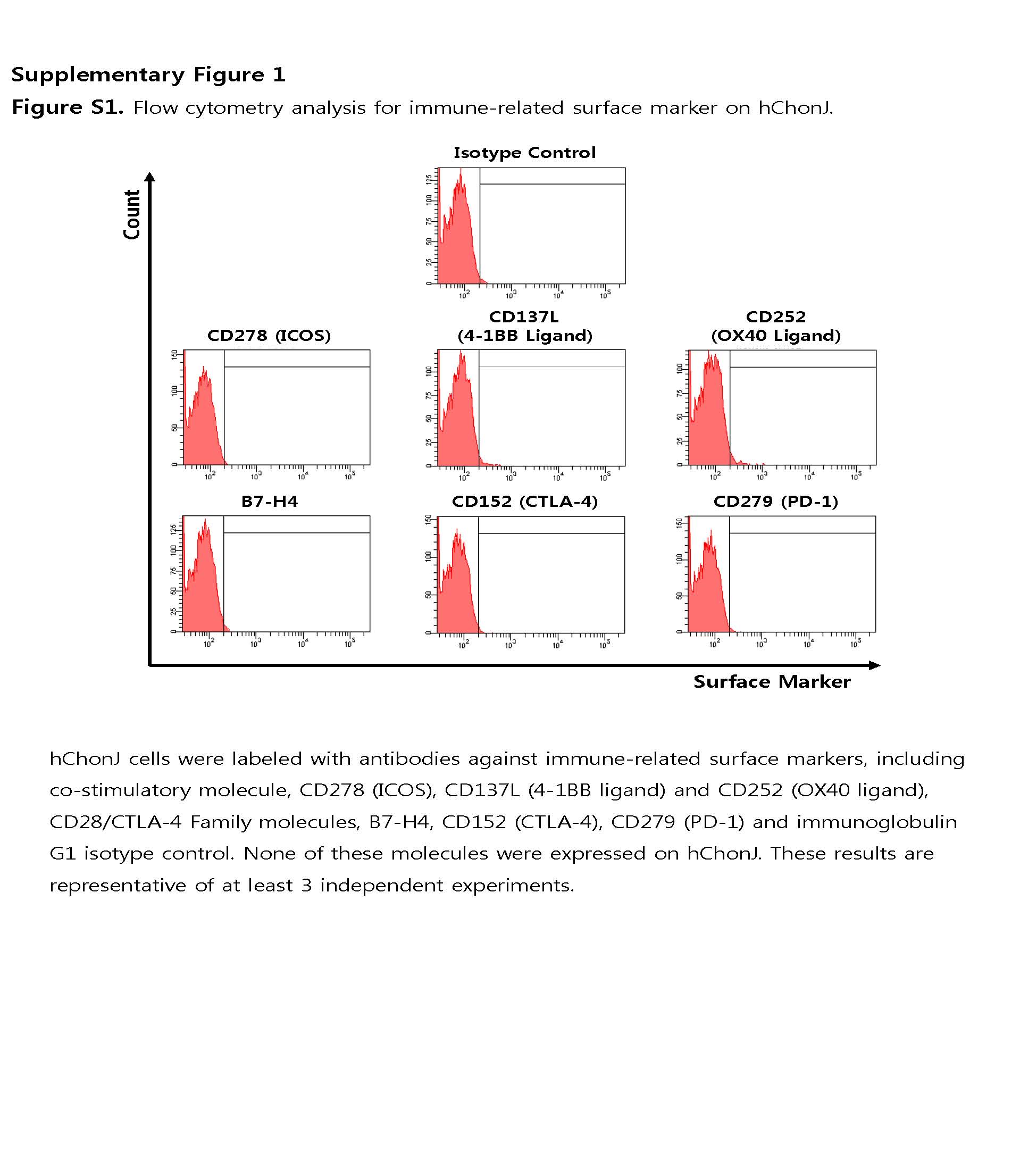

Supplement: Supplementary file 1 — Flow cytometry analysis for immune-related surface marker on hChonJ. (JPG 216 kb) [file 12891_2017_1547_MOESM1_ESM.jpg]

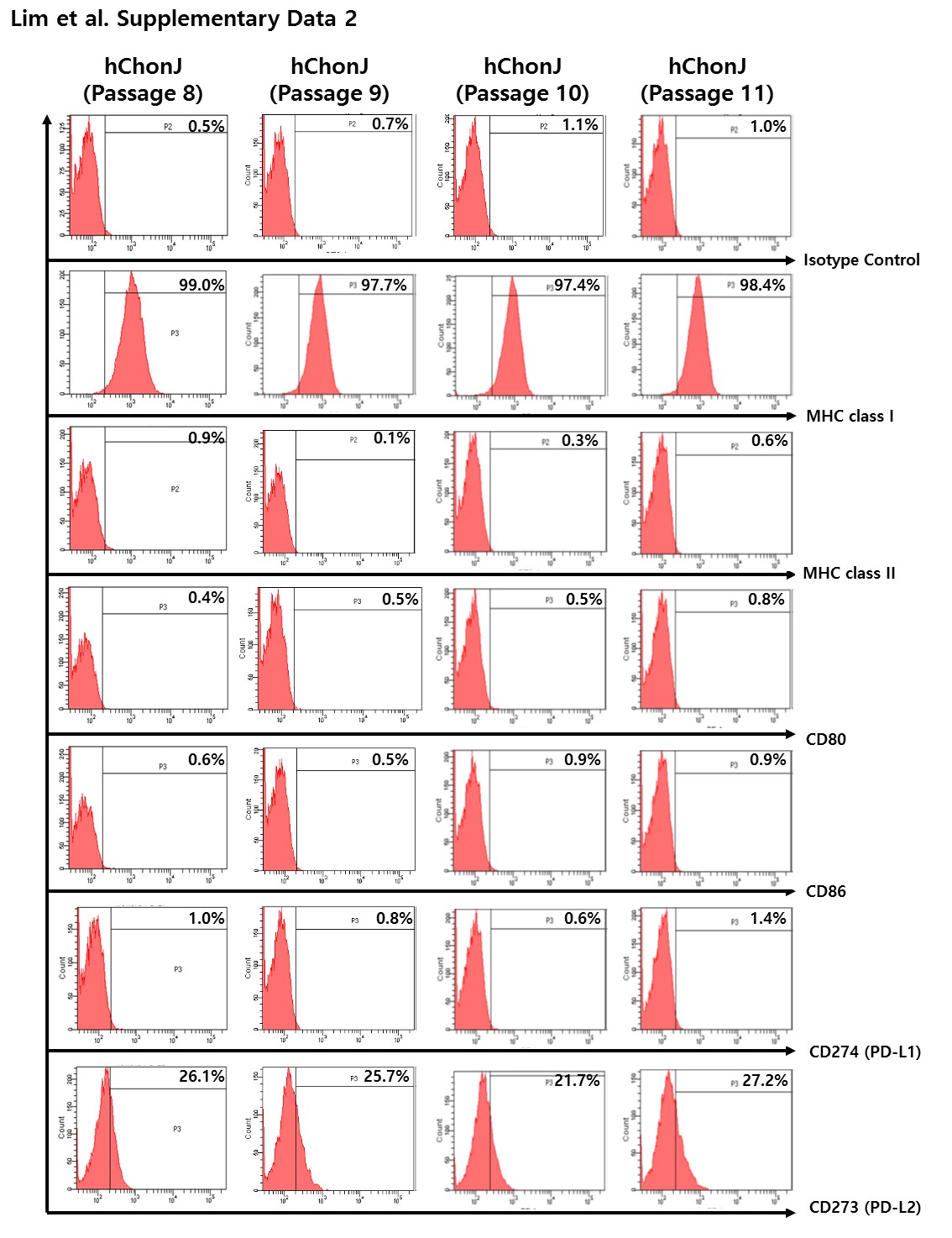

Supplement: Supplementary file 2 — Flow cytometry analysis for the expression pattern of MHC class I, MHC class II, co-stimulatory molecule (CD80, CD86) and co-inhibitory molecule (PD-L1, PD-L2) on 3 additional passaged hChonJ cells. (JPG 256 kb) [file 12891_2017_1547_MOESM2_ESM.jpg]
